# Pallidal Volumetry and Neutrophil-to-Lymphocyte Ratio in Differentiating Progressive Supranuclear Palsy from Parkinson’s Disease

**DOI:** 10.3390/diseases14090330

**Published:** 2026-09-09

**Authors:** Bartosz Migda, Michał Kutyłowski, Natalia Madetko-Alster, Anna Migda, Karol Kutyłowski, Piotr Alster

**Affiliations:** 1 Diagnostic Ultrasound Lab, Department of Pediatric Radiology, Medical University of Warsaw, 03-242 Warsaw, Poland; 2 Department of Radiology, Mazovian Brodno Hospital, 03-242 Warsaw, Poland; michael.kutylowski@gmail.com; 3 Department of Neurology, Medical University of Warsaw, 03-242 Warsaw, Poland; natalia.madetko@wum.edu.pl (N.M.-A.); karol.kutylowski@gmail.com (K.K.); piotr.alster@wum.edu.pl (P.A.); 4 Department of Endocrinology, Diabetology and Internal Medicine, Medical University of Warsaw, 03-242 Warsaw, Poland; anna.migda@wum.edu.pl

**Keywords:** progressive supranuclear palsy, Parkinson’s disease, pallidal volumetry, magnetic resonance imaging, neutrophil-to-lymphocyte ratio, biomarkers

## Abstract

Background: Progressive supranuclear palsy (PSP) and Parkinson’s disease (PD) frequently present with overlapping clinical features, creating diagnostic challenges, particularly during the early stages of disease. Structural MRI and peripheral inflammatory biomarkers may provide complementary information for differential diagnosis. Objective: To evaluate the diagnostic performance of automated pallidal volumetry and the neutrophil-to-lymphocyte ratio (NLR), individually and in combination, for distinguishing PSP from PD. Methods: This retrospective case–control study included 12 patients with PSP and 12 patients with PD. Automated brain volumetry was performed using the volBrain 2.0 platform. Total pallidal volume was selected as the primary imaging biomarker based on its established involvement in PSP pathology. NLR was calculated from routine blood counts. Diagnostic performance was assessed using receiver operating characteristic (ROC) analysis. A combined MRI–blood model was constructed using binary logistic regression. Results: Patients with PSP demonstrated lower normalized pallidal volumes than patients with PD. Pallidal volume showed high diagnostic performance for differentiating PSP from PD (AUC = 0.885), whereas NLR demonstrated only modest discriminatory ability (AUC = 0.715). The combined pallidal volume–NLR model achieved an AUC of 0.903 compared with 0.885 for pallidal volumetry alone, but this difference was not statistically significant (DeLong *p* = 0.732), and sensitivity, specificity and overall accuracy remained unchanged. Conclusions: Automated pallidal volumetry provided substantially better discrimination between PSP and PD than NLR alone. Although the combined model showed a small numerical increase in AUC, this improvement was not statistically significant and did not alter sensitivity, specificity or overall accuracy. These findings do not demonstrate incremental diagnostic value of NLR beyond pallidal volumetry in this cohort. Larger prospective studies are needed to determine the clinical utility of combined MRI–blood biomarker approaches in PSP.

## 1. Introduction

Parkinson’s disease (PD) and progressive supranuclear palsy (PSP) are neurodegenerative disorders characterized by distinct molecular pathologies but partially overlapping clinical phenotypes. PD is an α-synucleinopathy and represents one of the most common neurodegenerative disorders worldwide, with approximately 11.8 million affected individuals in 2021 [1]. In contrast, PSP is a rare primary 4-repeat tauopathy, with a pooled prevalence estimated at approximately 6.9 per 100,000 population, although reported estimates vary considerably depending on case ascertainment and diagnostic criteria [2,3,4].

PSP encompasses a heterogeneous spectrum of clinical phenotypes. The classical Richardson syndrome is characterized by early postural instability and falls, vertical supranuclear gaze palsy or slowing of vertical saccades, axial-predominant rigidity, bradykinesia, dysarthria, dysphagia, and frontal cognitive or behavioral dysfunction [2,3,5]. However, PSP-parkinsonism (PSP-P) may initially resemble PD, with bradykinesia, rigidity, asymmetric motor involvement, tremor and an initial response to levodopa [5,6]. This overlap is particularly relevant early in the disease course, before characteristic oculomotor abnormalities become evident. Early postural instability and unexplained falls, slowing of vertical saccades, axial rigidity, impaired postural reflexes, frontal-executive dysfunction and a poor or transient levodopa response should raise suspicion of PSP rather than idiopathic PD [2,3,5,6].

Neuroimaging may provide important supportive information in this differential diagnosis. Conventional structural MRI in PD is frequently normal or demonstrates relatively nonspecific abnormalities, and its principal clinical role remains the exclusion of structural causes of parkinsonism and identification of features suggesting an atypical parkinsonian disorder [7,8]. In PSP, by contrast, characteristic structural abnormalities include midbrain and superior cerebellar peduncle atrophy, an increased pons-to-midbrain ratio, and the visually recognizable “hummingbird” or “morning glory” signs [3,7,9,10,11,12]. Quantitative measures such as the Magnetic Resonance Parkinsonism Index (MRPI) and MRPI 2.0 can further improve discrimination between PSP and PD [10,13,14,15]. Nevertheless, visual MRI signs have limited sensitivity, particularly at early disease stages and in PSP-P, and overlap between diagnostic groups remains an important limitation [12].

Beyond conventional planimetric measures, quantitative MRI has demonstrated more extensive patterns of PSP-related neurodegeneration involving the brainstem, basal ganglia and frontal-subcortical networks [3,9,10,11,16,17]. The globus pallidus is consistently affected by PSP-related tau pathology and volumetric studies have demonstrated discriminatory value of pallidal and other subcortical measurements in differentiating PSP from PD [3,11,16,17]. Automated volumetric approaches may therefore provide complementary quantitative information beyond conventional visual assessment and reduce dependence on observer-defined measurements.

The biomarker landscape of parkinsonian disorders is also expanding beyond structural imaging. α-Synuclein seed amplification assays provide a pathology-oriented approach to identifying synucleinopathy, while neurofilament light chain, tau-related measures, molecular imaging and other fluid biomarkers are being investigated for differential diagnosis and disease characterization [18,19,20]. However, many of these techniques require specialized laboratory platforms, cerebrospinal fluid sampling or advanced imaging and are not yet routinely available for differential diagnosis of PSP and PD.

In this context, readily available peripheral inflammatory indices may represent a simple complementary source of biological information. The neutrophil-to-lymphocyte ratio (NLR), derived from routine complete blood counts, has been investigated as a marker of systemic immune activation in PD and atypical parkinsonian syndromes [21,22,23,24]. Elevated NLR has been reported in PSP, although substantial overlap between diagnostic groups and sensitivity to systemic confounders limit its value as a stand-alone diagnostic marker [21,22,24]. Rather than replacing established clinical, imaging or molecular biomarkers, NLR may therefore be considered a readily accessible exploratory marker whose potential value lies in combination with more disease-relevant measures.

Because structural MRI and peripheral inflammatory markers reflect different aspects of disease biology, the present study evaluated the diagnostic performance of automated pallidal volumetry and NLR, individually and in combination, for differentiating PSP from PD.

## 2. Materials and Methods

### 2.1. Study Population

This retrospective case–control study included patients with progressive supranuclear palsy (PSP) and Parkinson’s disease (PD) evaluated at the Department of Neurology, Medical University of Warsaw and identified from the institutional database between January 2024 and December 2025. A total of 34 patients with parkinsonism were retrospectively screened for eligibility. Ten patients were excluded because of extensive cerebrovascular pathology (n = 4), intracranial neoplasm (n = 3), major structural abnormalities unrelated to neurodegeneration (n = 2), or imaging artifacts precluding reliable volumetric analysis (n = 1). The final cohort included 12 patients with PD (9 males, 75.0%; median age 72.0 years [IQR 66.0–73.0]) and 12 patients with PSP (7 males, 58.3%; median age 72.0 years [IQR 67.0–74.0]). All participants included in the final analysis had both MRI suitable for automated volumetric analysis and available CBC data; therefore, no missing MRI or blood data were present in the analyzed cohort.

The study was conducted in accordance with the Declaration of Helsinki and was approved by the Bioethics Committee of the Medical University of Warsaw (approval no. AKBE/243/2016; 13 December 2016). The present study was based on retrospective analysis of previously collected clinical and imaging data. Written informed consent for the use of clinical and imaging data for research purposes had been obtained from all participants.

**Figure 1:**
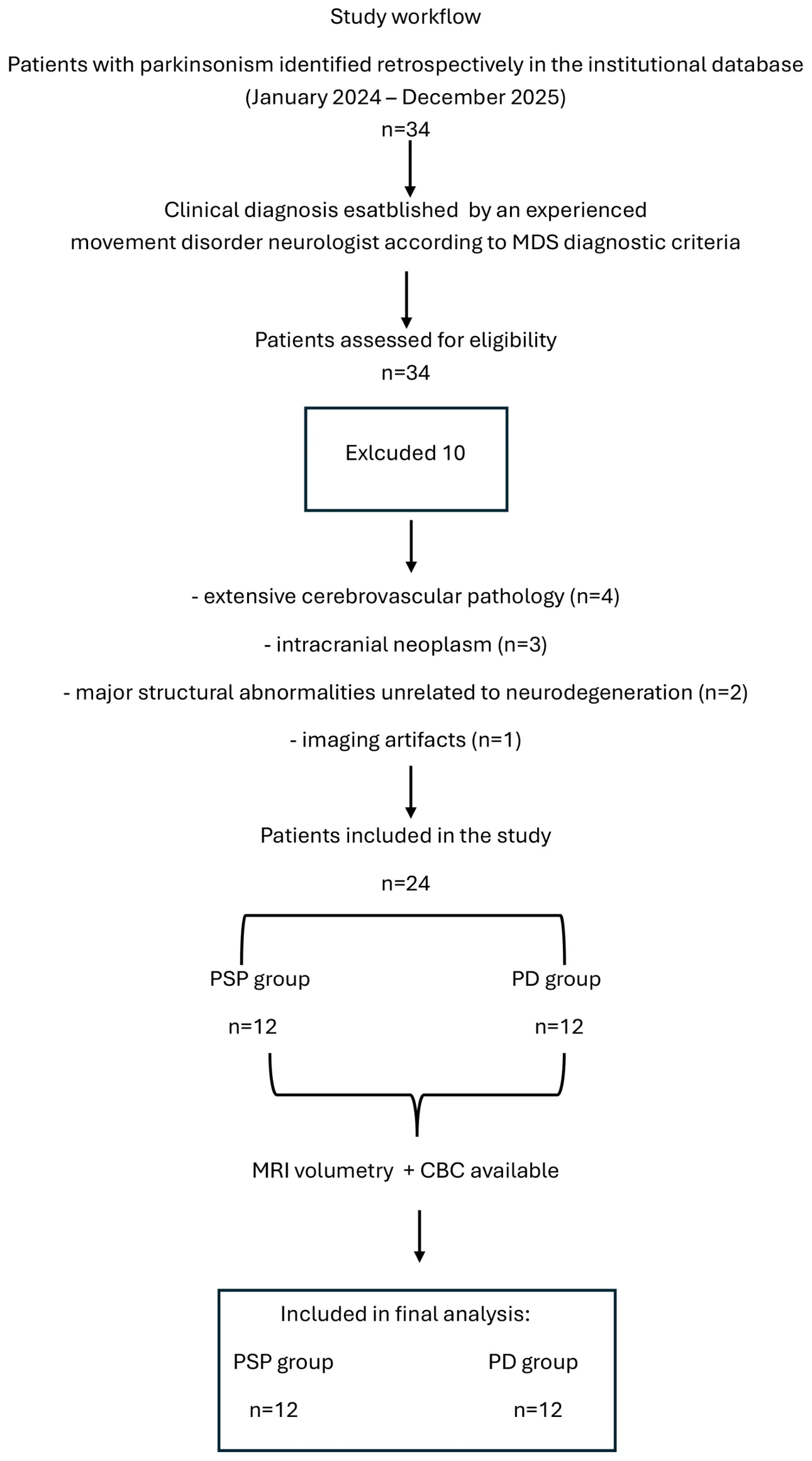
Flow diagram of retrospective patient identification, eligibility assessment, exclusions, and inclusion in the final analysis.

All clinical diagnoses were established by an experienced movement-disorder neurologist according to the respective MDS diagnostic criteria [2,25]. The neurologist did not have access to the MRI results used for the present study at the time of clinical diagnostic classification. Automated volBrain analysis was subsequently performed in the final cohort of 24 patients and therefore did not contribute to the clinical diagnosis. The patient selection process is summarized in Figure 1.

### 2.2. Peripheral Inflammatory Biomarkers

Peripheral inflammatory status was assessed using routine hematological parameters obtained from venous blood samples collected during the diagnostic work-up.

The primary inflammatory biomarker analysed in the present study was the neutrophil-to-lymphocyte ratio (NLR), calculated by dividing the absolute neutrophil count by the absolute lymphocyte count. NLR was selected a priori because previous studies have demonstrated its potential relevance in atypical parkinsonian syndromes and PSP [23,24]. The exact interval between blood collection and MRI examination was not available due to the retrospective design of the study.

### 2.3. MRI Acquisition and Automated Volumetric Analysis

MRI examinations were performed using a 3-T Siemens MAGNETOM Skyra system (Siemens Healthineers, Erlangen, Germany) equipped with a 20-channel head coil. All patients were examined using the same scanner and standardized clinical MRI protocol. High-resolution three-dimensional T1-weighted images were acquired using a Magnetization Prepared Rapid Gradient Echo (MPRAGE) sequence with the following parameters: echo time (TE) = 2.6 ms, repetition time (TR) = 1540 ms, inversion time (TI) = 800 ms, flip angle = 8°, field of view (FOV) = 260 × 320 mm^2^, acquisition matrix = 260 × 320, and isotropic spatial resolution of 1 mm.

**Figure 2:**
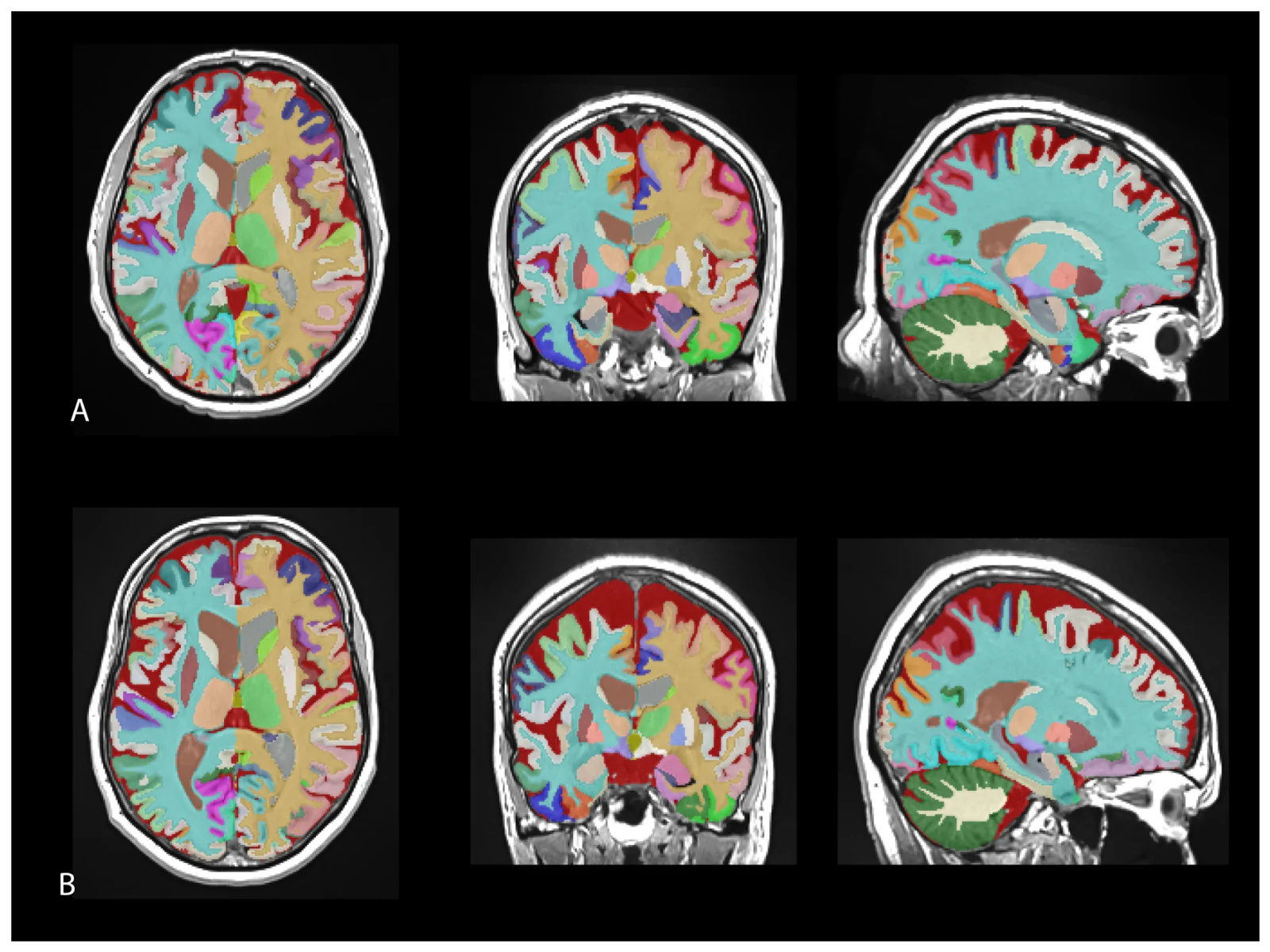
Representative examples of automated brain segmentation using the volBrain 2.0 platform and the vol2Brain pipeline. (**A**) Patient with Parkinson’s disease (PD) and (**B**) patient with progressive supranuclear palsy (PSP). Color-coded segmentation overlays are presented on T1-weighted MR images in axial, coronal, and sagittal planes, illustrating the automated delineation of cortical and subcortical brain structures used for subsequent volumetric analysis.

Automated volumetric assessment was performed using the volBrain 2.0 platform and the vol2Brain segmentation pipeline, version 1.0 (release 23 November 2021) [26,27]. The processing workflow includes tissue classification, anatomical parcellation, multi-atlas segmentation, and automated correction procedures, providing reproducible measurements of cortical and subcortical brain structures. All segmentation reports were visually reviewed by a neuroradiologist who was blinded to both patient identity and clinical diagnosis. No segmentation failures occurred, and no cases were excluded because of segmentation quality. Representative vol2Brain segmentation results from patients with PD and PSP are presented in Figure 2.

Although vol2Brain provides volumetric measurements for a substantially larger number of anatomical structures, the present hypothesis-driven analysis was restricted to eight regional measures selected for their relevance to the cortical–subcortical networks implicated in PSP (SFG volume, pallidum volume, frontal volume, caudate volume, brainstem volume, subcortical GM volume, putamen volume, thalamus volume). The remaining vol2Brain outputs were not screened as candidate biomarkers in the present analysis.

Based on current neuropathological and neuroimaging evidence indicating prominent pallidal involvement in PSP [3,11,16,17], particular attention was directed towards pallidal volumetry. Total pallidal volume and hemispheric pallidal volumes were extracted from the automated segmentation output. All volumetric measurements were normalized to intracranial volume and expressed as percentages of intracranial volume to minimize inter-individual variability related to head size. For the final analysis, total pallidal volume was selected before examination of the volumetric results of the present cohort as the primary MRI variable for the multimodal analysis. This selection was hypothesis-driven and based on the established involvement of the pallidum in PSP-related subcortical neurodegeneration rather than on its observed discriminatory performance in the present dataset.

### 2.4. Statistical Analysis

Statistical analyses were performed using Statistica version 13 (TIBCO Software Inc., Palo Alto, CA, USA). Continuous variables were assessed for normality using the Shapiro–Wilk test. No a priori sample size calculation was performed because of the retrospective and exploratory study design; the sample size was determined by the number of eligible patients available in the study database. Because most variables were not normally distributed and the study groups were relatively small, non-parametric methods were applied for descriptive and between-group analysis. Continuous variables are presented as median and interquartile range (IQR), whereas categorical variables are reported as counts and percentages. Comparisons between PSP and PD were performed using the Mann–Whitney U test for continuous variables and Fisher’s exact test for categorical variables.

For the regional volumetric comparisons the Benjamini–Hochberg false discovery rate correction was applied across the eight prespecified regional comparisons (n = 8). For the secondary ROC analysis, the five MRI regions showing the strongest between-group differences were carried forward for evaluation of diagnostic performance.

The diagnostic performance of individual biomarkers was assessed using receiver operating characteristic (ROC) analysis. Areas under the ROC curve (AUCs), corresponding 95% confidence intervals, and *p*-values testing the null hypothesis of AUC = 0.5 were obtained using the ROC analysis procedure implemented in Statistica version 13. These ROC-derived *p*-values are distinct from the *p*-values obtained from direct between-group comparisons using the Mann–Whitney U test. Optimal cut-off values were determined using the Youden index. Because multiple candidate biomarkers were evaluated in parallel, *p*-values derived from the 12 ROC analyses (five MRI measures and seven hematological/inflammatory measures) were adjusted using the Benjamini–Hochberg false discovery rate (FDR) procedure across all 12 tests (m = 12). Both unadjusted *p*-values and FDR-adjusted q-values are reported. The AUC of the combined pallidal volume NLR model was formally compared with the AUC of pallidal volumetry alone using the non-parametric DeLong test for correlated ROC curves.

To investigate whether combining imaging and inflammatory biomarkers improved diagnostic discrimination, binary logistic regression models incorporating pallidal volume and neutrophil-to-lymphocyte ratio (NLR) were constructed. Predicted probabilities generated by the logistic regression model were subsequently used for ROC analysis. Given the limited sample size, internal validation of the combined pallidal volume–NLR logistic regression model was performed using non-parametric bootstrap resampling with 2000 repetitions. In each bootstrap sample, the model was refitted, and its AUC was calculated both in the bootstrap sample and after application to the original dataset. Optimism was estimated as the mean difference between these two AUC values, and the optimism-corrected AUC was obtained by subtracting the estimated optimism from the apparent AUC. Regression coefficients, standard errors, odds ratios (ORs), 95% confidence intervals and *p*-values were additionally reported for the complete logistic regression model. Because threshold selection and the resulting sensitivity, specificity and accuracy were derived from the development cohort, these measures were considered apparent in-sample estimates.

All statistical tests were two-sided and *p*-values below 0.05 were considered statistically significant.

## 3. Results

### 3.1. Demographic and Inflammatory Characteristics

The PSP and PD groups did not differ significantly in age (*p* = 0.6636) or sex distribution (75% male in PD vs. 58.3% in PSP; Fisher exact test, *p* = 0.667). Although NLR values tended to be higher in PSP, the difference between groups did not reach statistical significance (Table 1).

### 3.2. MRI Volumetric Differences Between PSP and PD

**Figure 3:**
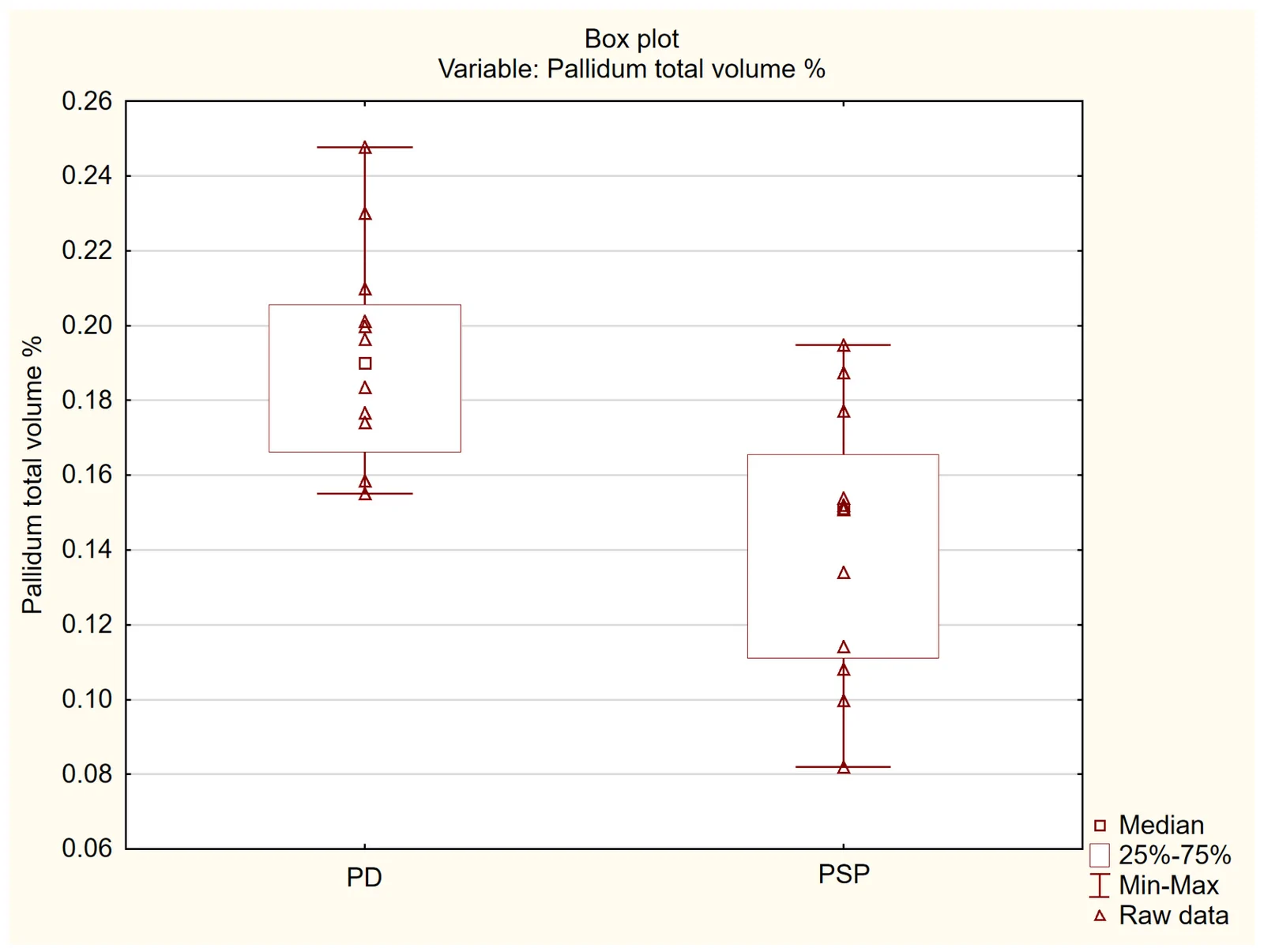
Distribution of normalized total pallidal volume in patients with Parkinson’s disease and progressive supranuclear palsy. Box plots present the median, interquartile range, and minimum–maximum values; triangles represent raw data. Pallidal volumes are expressed as percentages of intracranial volume. PD, Parkinson’s disease; PSP, progressive supranuclear palsy.

Among the analysed volumetric parameters, several regions demonstrated significant between-group differences. Because pallidal degeneration was selected a priori as the primary imaging biomarker based on its established role in PSP pathology, subsequent analyses focused on pallidal volumetry. Pallidal volume was significantly lower in PSP than in PD (median 0.151% ICV [IQR 0.111–0.166] vs. 0.190% ICV [IQR 0.166–0.205], respectively; *p* = 0.00165, FDR-adjusted q = 0.0066 (Table 2). Figure 3 illustrates the distribution of pallidal volumes in both groups.

### 3.3. Diagnostic Performance of Individual Biomarkers

**Figure 4:**
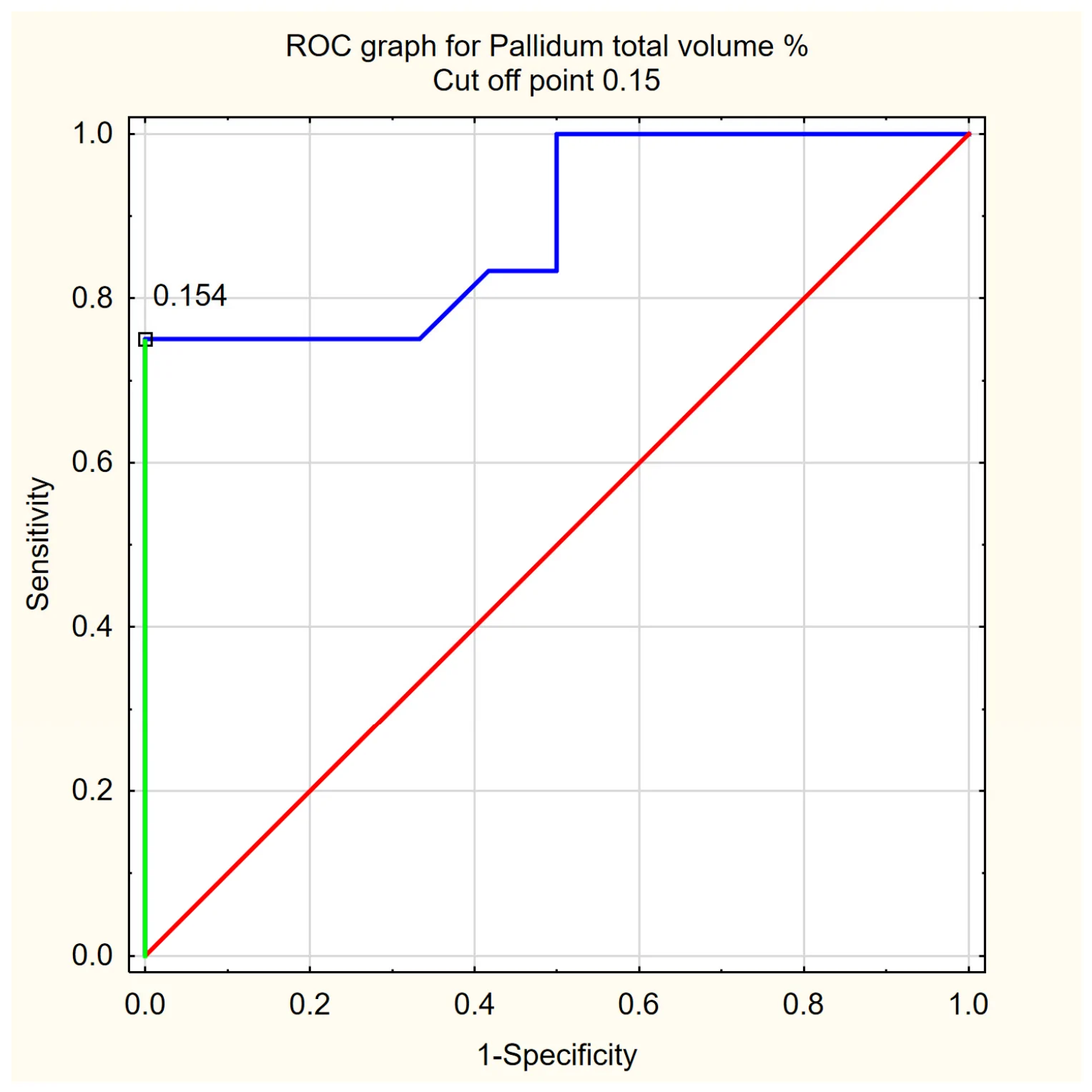
Receiver operating characteristic curve for normalized total pallidal volume in differentiating progressive supranuclear palsy from Parkinson’s disease. The area under the curve was 0.885, with an optimal cut-off value of ≤0.154% of intracranial volume, sensitivity of 75.0%, and specificity of 100.0%. The optimal threshold was determined using the Youden index.

**Figure 5:**
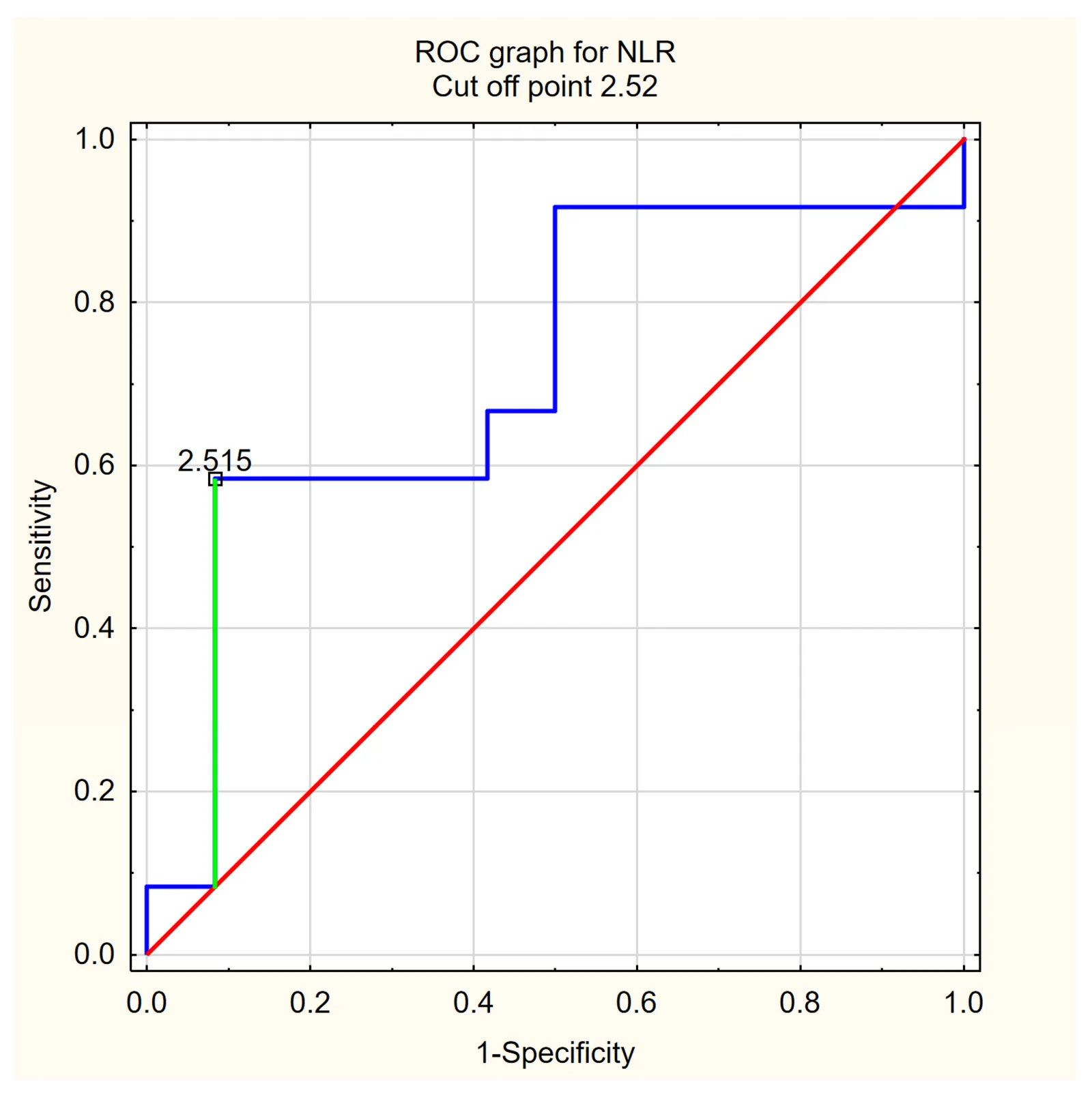
Receiver operating characteristic curve for the neutrophil-to-lymphocyte ratio in differentiating progressive supranuclear palsy from Parkinson’s disease. The area under the curve was 0.715, with an optimal cut-off value of ≥2.515, sensitivity of 58.3%, and specificity of 91.7%. The optimal threshold was determined using the Youden index. NLR, neutrophil-to-lymphocyte ratio.

ROC analysis demonstrated high diagnostic performance of pallidal volumetry for differentiating PSP from PD (AUC = 0.885). Because pallidal degeneration represents a well-established neuropathological hallmark of PSP and was selected a priori as the primary imaging biomarker, subsequent combined modelling focused on pallidal volume and NLR. NLR alone showed only modest discriminatory ability (AUC = 0.715). Detailed ROC parameters are presented in Table 3. ROC curves for pallidal volume and NLR are shown in Figure 4 and Figure 5, respectively.

**Figure 6:**
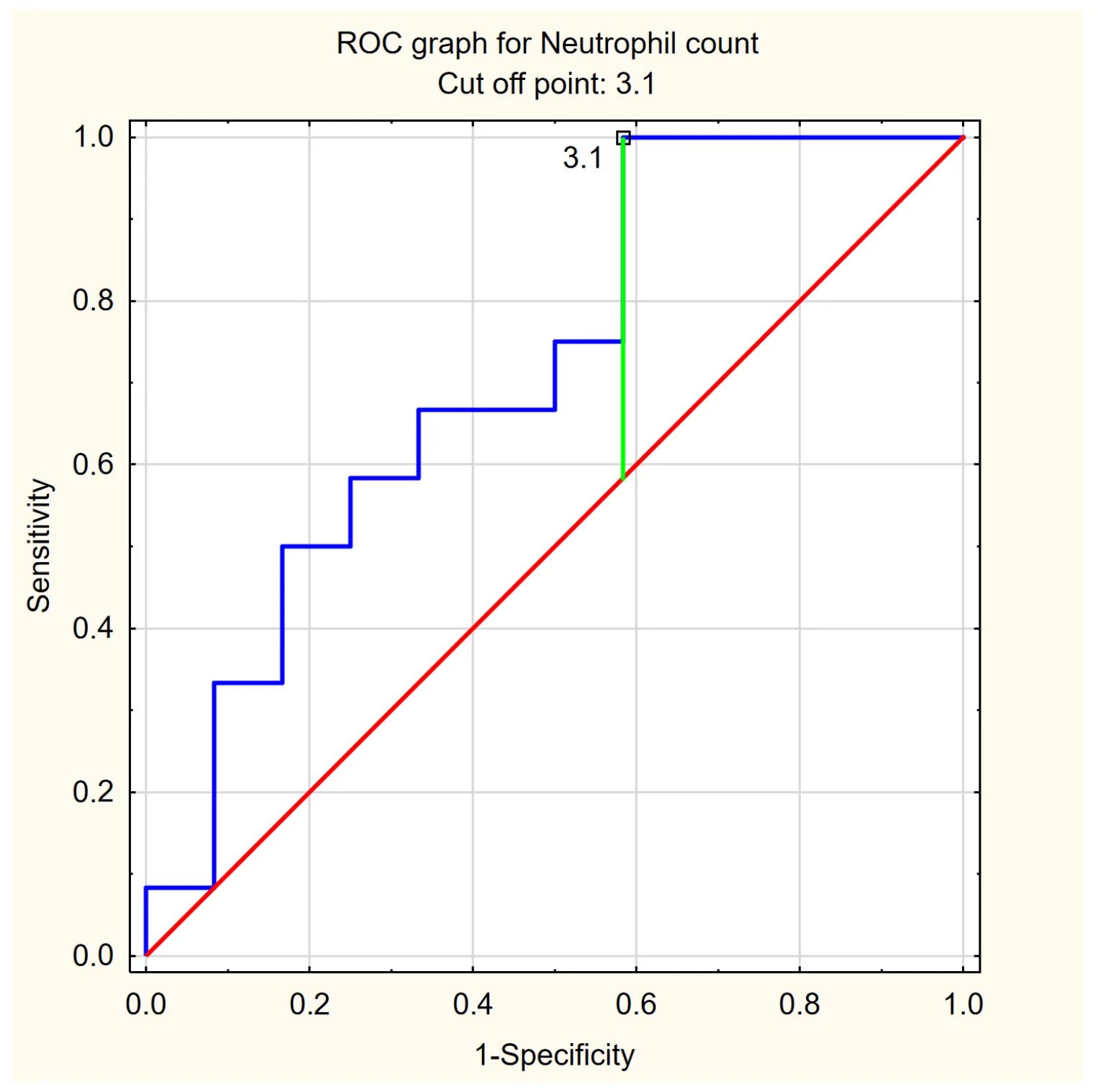
Receiver operating characteristic curve for neutrophil count in differentiating progressive supranuclear palsy from Parkinson’s disease. The area under the curve was 0.715, with an optimal cut-off value of ≥3.10 × 10^9^/L, sensitivity of 100.0%, and specificity of 47.1%. The optimal threshold was determined using the Youden index.

Because lymphocyte counts were similar between groups, whereas neutrophil counts were numerically higher in PSP, an additional exploratory ROC analysis was performed for absolute neutrophil count (Table 3, Figure 6). Neutrophil count yielded an AUC of 0.715, identical to that observed for NLR. Neutrophil counts were numerically higher in PSP than in PD (Table 2), although the difference did not reach statistical significance (*p* = 0.0783).

### 3.4. Combined MRI–Blood Biomarker Model

To evaluate whether inflammatory information provided additional diagnostic value beyond MRI volumetry, a combined logistic regression model incorporating pallidal volume and NLR was constructed.

**Figure 7:**
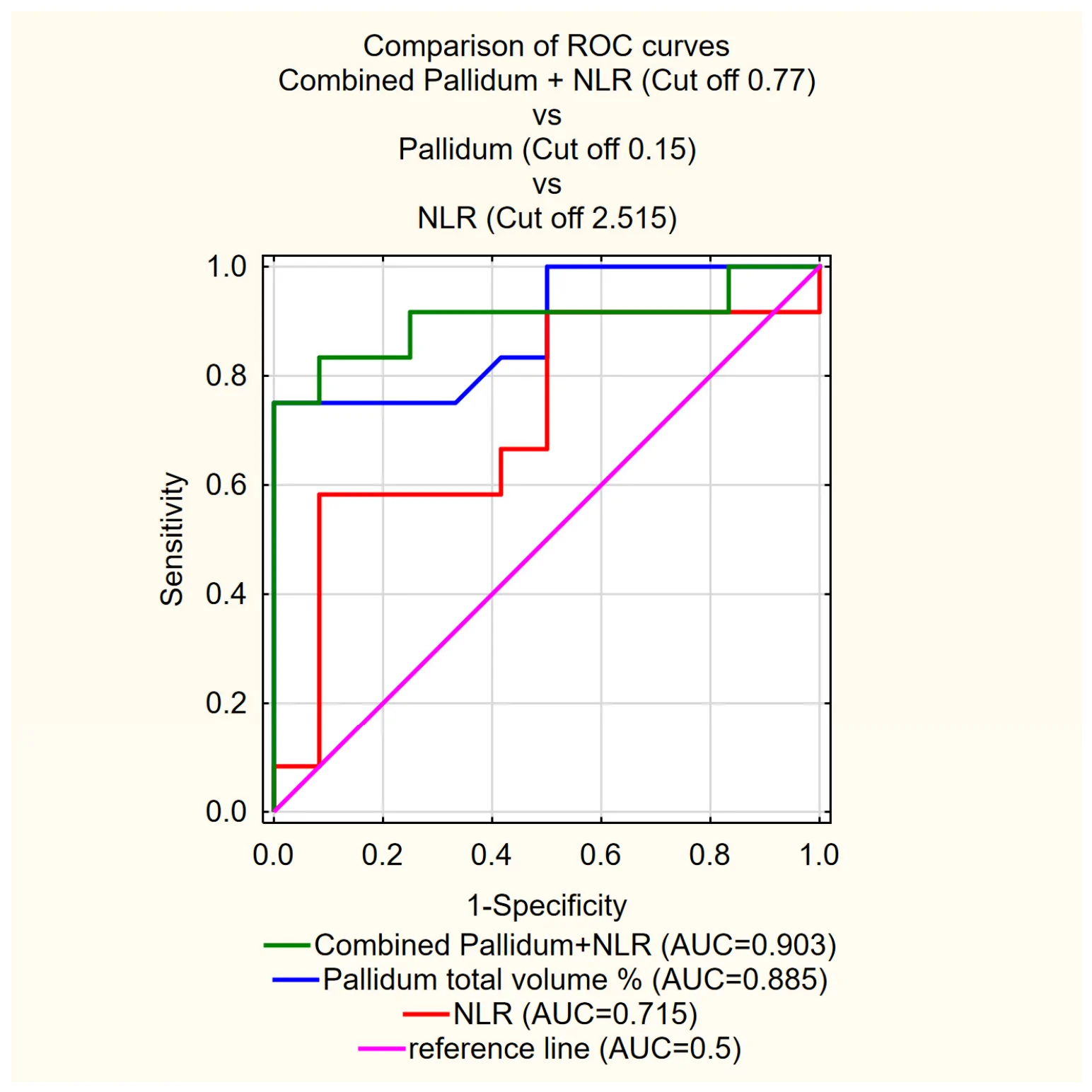
Comparison of receiver operating characteristic curves for normalized total pallidal volume, neutrophil-to-lymphocyte ratio, and the combined pallidal volume–NLR logistic regression model in differentiating progressive supranuclear palsy from Parkinson’s disease. The areas under the curve were 0.885 for pallidal volume, 0.715 for NLR, and 0.903 for the combined model. The combined model yielded a marginal improvement over pallidal volumetry alone. NLR, neutrophil-to-lymphocyte ratio.

The combined model yielded an apparent AUC of 0.903 compared with 0.885 for pallidal volumetry alone (Table 4, Figure 7). This numerical difference was not statistically significant (DeLong test, *p* = 0.732). Bootstrap internal validation (2000 bootstrap resamples) yielded an optimism-corrected AUC of 0.879 for the combined model. The apparent optimal probability threshold was 0.771, corresponding to a sensitivity of 75.0%, specificity of 100.0% and overall accuracy of 87.5% in the development cohort, and remained unchanged after additional NLR. These threshold-based measures should be interpreted as apparent in-sample estimates (Table 4).

The fitted logistic regression model was logit(PPSP) = 5.786 − 59.284 × pallidum total volume (%) + 1.787 × NLR. Pallidal volume was significantly associated with PSP classification (β = −59.284, SE = 25.438, *p* = 0.0198). Expressed per 0.01 percentage-point increase in normalized pallidal volume, the corresponding OR was 0.553 (95% CI, 0.336–0.910). NLR showed a positive, but not statistically significant, association with PSP classification (β = 1.787, SE = 1.016; OR = 5.97, 95% CI, 0.81–43.75; *p* = 0.0788; Table 5).

The reported cut-off values were derived and evaluated in the same cohort and were therefore considered exploratory cohort-specific thresholds. Ninety-five-percent confidence intervals were calculated for sensitivity, specificity, and accuracy (Table S1).

## 4. Discussion

This study examined whether pallidal volumetry and the neutrophil-to-lymphocyte ratio (NLR) could improve differentiation between progressive supranuclear palsy (PSP) and Parkinson’s disease (PD). The main discriminatory signal came from MRI: pallidal volume differentiated PSP from PD better than NLR alone. Adding NLR produced only a small numerical increase in AUC, which was not statistically significant and did not improve sensitivity, specificity or accuracy. Thus, the present data do not demonstrate incremental diagnostic value of NLR beyond pallidal volumetry. The reduction in pallidal volume in PSP is biologically plausible. The globus pallidus is preferentially affected by 4-repeat tau pathology, together with the subthalamic nucleus, substantia nigra, brainstem and frontal-subcortical circuits [3,16,17]. MRI studies have demonstrated neostriatal and mesencephalic atrophy as well as progressive subcortical and cortical involvement across PSP variants [16,17]. Pallidal volumetry was therefore prespecified as a biologically grounded imaging marker of PSP-related neurodegeneration rather than selected on the basis of its performance in the present cohort.

Notably, superior frontal gyrus (SFG) volume showed the highest discriminatory performance among the regional measures examined in this cohort (AUC = 0.927), numerically exceeding that of pallidal volume (AUC = 0.885). This finding is consistent with previous MRI studies demonstrating prominent frontal cortical involvement, including superior frontal abnormalities, in PSP [16,28]. However, SFG was not included in the primary combined model because pallidal volume had been prespecified as the primary MRI marker before examination of the volumetric results of the present cohort. Selecting SFG for model construction on the basis of its observed AUC would therefore constitute post hoc, data-driven predictor selection, with a substantial risk of optimistic performance estimates in this small sample. The diagnostic value of SFG volumetry should be evaluated prospectively in an independent cohort.

Pallidal atrophy should not, however, be treated as a disease-specific marker. Volume loss in basal ganglia structures may also occur in multiple system atrophy, advanced PD and other neurodegenerative conditions [11,17]. Differences between studies may additionally result from disease stage, phenotype composition, MRI acquisition, segmentation method, and whether manual, atlas-based or automated volumetry was used. The relatively clear separation observed here may therefore reflect the inclusion of clinically established cases rather than performance expected in early or diagnostically uncertain parkinsonism.

The inflammatory component of the analysis was less decisive. NLR was numerically higher in PSP, but the difference did not reach statistical significance, consistent with previous studies showing substantial overlap of peripheral inflammatory indices across parkinsonian syndromes [21,22,23,24]. Notably, the modest NLR signal in the present cohort appeared to be driven predominantly by neutrophils: lymphocyte counts were similar between PSP and PD, whereas neutrophil counts were numerically higher in PSP. Neutrophil count alone yielded an AUC of 0.715, identical to that of NLR, although neither difference was statistically significant. This pattern is consistent with previous observations in PSP [22,24].

MRI volumetry and NLR reflect different biological processes. Pallidal volume represents cumulative structural neurodegeneration, whereas NLR is a dynamic systemic marker influenced by infection, metabolic status, vascular comorbidity and medication use. A single blood measurement may therefore only weakly reflect the chronic neurodegenerative process, which may partly explain the limited diagnostic contribution of NLR in this cohort. The combined MRI–blood model yielded the numerically highest AUC; however, the increase relative to pallidal volumetry alone was small and not statistically significant. Accordingly, the present findings do not establish incremental diagnostic value of NLR. Nevertheless, multimodal biomarker strategies remain relevant in PSP, where imaging, fluid biomarkers, clinical measures and molecular markers may provide complementary information [11,18,19]. Whether peripheral inflammatory markers can contribute meaningfully to such approaches requires evaluation in larger independent cohorts.

Whether NLR reflects mechanisms directly involved in PSP remains uncertain. Evidence from TSPO PET studies supports the presence of neuroinflammation in PSP and suggests spatial overlap with regions affected by tau pathology in primary 4-repeat tauopathies [18,19]. Analyses examining the association between in vivo tau distribution PET support TSPO PET as a marker related to microglial activation [29]. Recent neuropathological data also indicate that adaptive immune mechanisms may be involved, with cytotoxic T-cell infiltration reported in vulnerable midbrain regions in PSP [18]. These findings support a role for immune activation in PSP, but they do not establish that peripheral inflammatory indices directly mirror central neuroinflammatory processes.

Despite this growing body of evidence, the relationship between central neuroinflammation and peripheral inflammatory indices remains poorly understood. Blood-derived markers such as NLR represent systemic immune activity and may not directly mirror inflammatory events occurring within the central nervous system. This biological dissociation may explain why convincing evidence for neuroinflammation in PSP does not necessarily translate into strong diagnostic performance of peripheral inflammatory markers in individual patients.

Several published studies argue for caution. Peripheral inflammatory indices have shown overlap between PSP, PD and other parkinsonian syndromes [21,23,24]. In PD, NLR and related indices have been linked to disease severity, motor phenotype and other clinical features rather than to diagnosis alone [30,31,32,33,34,35]. Thus, an elevated inflammatory index may reflect general neurodegenerative burden, systemic vulnerability or comorbidity rather than a PSP-specific process. This is particularly relevant in small retrospective cohorts, where a few individual values can influence model performance.

The clinical appeal of the proposed model lies in its simplicity and potential accessibility. Automated MRI volumetry can be performed using routine high-resolution T1-weighted sequences that are already included in standard neuroimaging protocols for patients with parkinsonian syndromes. Similarly, NLR is derived from a routinely available complete blood count and does not require specialized laboratory techniques. From a practical perspective, this makes the proposed approach substantially more accessible than advanced molecular imaging or cerebrospinal fluid biomarkers.

At present, however, the findings should be regarded as exploratory rather than practice-changing. The improvement achieved by adding NLR to pallidal volumetry was relatively small and would not be expected to alter clinical decision-making in patients with established PSP or PD. The potential value of such multimodal models may emerge in diagnostically challenging situations, particularly during earlier disease stages when structural changes are less pronounced and clinical features remain ambiguous. Future studies should therefore evaluate combined imaging and blood-based biomarkers in broader cohorts that include early PSP, uncertain parkinsonism, multiple system atrophy, corticobasal syndrome and healthy controls. Demonstrating incremental value in these clinically relevant settings will be essential before implementation in routine diagnostic pathways can be considered.

## 5. Limitations

This study has several limitations. The cohort was small, no a priori sample size calculation was performed and the size was determined by the number of eligible patients available in the retrospective database, reflecting the rarity of PSP but limiting statistical precision and increasing susceptibility to sampling effects. The retrospective design did not allow standardized blood sampling, detailed control of medication status or systematic exclusion of transient inflammatory conditions. In addition, sufficiently complete and standardized clinical data on disease duration, age at onset, motor and cognitive severity, levodopa responsiveness, and medication exposure were not available for the entire cohort. Detailed retrospective classification into MDS-defined PSP predominance types was therefore not considered sufficiently reliable. Consequently, we cannot exclude the possibility that differences in disease stage or PSP phenotype contributed to the observed volumetric differences between the groups. PSP diagnoses were based on clinical criteria without neuropathological confirmation. Given the small sample size, the potential influence of sex on volumetric and inflammatory measures could not be reliably assessed and cannot be excluded.

Furthermore, the cut-off values and associated diagnostic performance estimates were derived from the same small cohort and should be regarded as exploratory rather than clinically validated thresholds. The balanced 1:1 PSP-to-PD group composition also differs from the prevalence encountered in clinical practice, limiting the generalizability of the reported accuracy.

The combined model should be interpreted as exploratory. The small sample size increases the risk of overfitting and may produce optimistic estimates of diagnostic performance. Bootstrap internal validation demonstrated only a modest reduction in discrimination, with the AUC decreasing from an apparent value of 0.903 to an optimism-corrected value of 0.879. However, internal resampling cannot substitute for evaluation in an independent cohort. In particular, the reported cut-off, sensitivity, specificity and accuracy were derived from the development dataset and should not be interpreted as externally validated operating characteristics. External validation in a substantially larger and independent cohort is therefore required before clinical application can be considered. Finally, only NLR was included as the inflammatory component; other immune markers may provide different or complementary information.

## 6. Conclusions

Automated pallidal volumetry showed potential diagnostic value for differentiating PSP from PD in this small cohort. Adding NLR produced only a small, non-significant increase in AUC and did not improve sensitivity, specificity, or accuracy. Therefore, the present study does not demonstrate incremental diagnostic value of NLR beyond pallidal volumetry. Larger independent cohorts are required to validate the diagnostic performance of pallidal volumetry and to determine whether peripheral inflammatory markers provide clinically meaningful additional information.

## Figures and Tables

**Table 1:** Demographic characteristics and peripheral inflammatory indices in patients with Parkinson’s disease (PD) and progressive supranuclear palsy (PSP).

Variable	PD n = 12	PSP n = 12	*p*
Age (years)	72.0 (66.0–73.0)	72.0 (67.0–74.0)	0.6636
Male sex, n (%)	9 (75.0%)	7 (58.3%)	0.667
NEU (×10^9^/L)	3.44 (2.61–4.21)	4.33 (3.36–5.17)	0.0782
LYM (×10^9^/L)	1.71 (1.28–1.95)	1.73 (1.46–2.19)	0.5253
MON (×10^9^/L)	0.59 (0.42–0.69)	0.54 (0.46–0.71)	0.7506
PLT (×10^9^/L)	224.0 (188.0–277.0)	219.5 (198.5–239.5)	0.9080
NLR	1.96 (1.73–2.34)	2.55 (2.03–3.04)	0.0783
PLR	127.13 (100.04–157.48)	132.21 (98.47–150.69)	0.7950
MLR	0.351 (0.261–0.378)	0.300 (0.284–0.381)	0.6236
SIRI (×10^9^/L)	1.31 (0.87–1.47)	1.39 (0.99–1.88)	0.2366
SII (×10^9^/L)	483.58 (352.3–571.2)	570.28 (420.47–696.62)	0.1409
RDW-CV (%)	12.35 (12.05–13.05)	13.00 (12.25–13.6)	0.2353

Abbreviations: NEU, neutrophil count; LYM, lymphocyte count; MON, monocyte count; PLT, platelet count; NLR, neutrophil-to-lymphocyte ratio; PLR, platelet-to-lymphocyte ratio; MLR, monocyte-to-lymphocyte ratio; SIRI, systemic inflammation response index; SII, systemic immune-inflammation index; RDW-CV, red blood cell distribution width coefficient of variation. Note: Continuous variables are presented as median (interquartile range, IQR) and categorical variables as number (percentage). Group comparisons were performed using the Mann–Whitney U test or Fisher’s exact test, as appropriate.

**Table 2:** Comparison of normalized MRI-derived regional brain volumes between patients with Parkinson’s disease (PD) and progressive supranuclear palsy (PSP).

MRI Variable	PD Median (IQR)	PSP Median (IQR)	*p*	FDR q
SFG total volume %	2.003 (1.955–2.073)	1.766 (1.705–1.872)	0.00048	0.0038
Pallidum total volume %	0.190 (0.166–0.205)	0.151 (0.111–0.166)	0.00165	0.0066
Frontal total volume %	11.423 (11.199–11.864)	11.070 (10.688–11.407)	0.0351	0.081
Caudate total volume %	0.475 (0.463–0.525)	0.440 (0.401–0.486)	0.0404	0.081
Brainstem volume %	1.350 (1.242–1.499)	1.239 (1.171–1.326)	0.0605	0.097
Subcortical GM volume %	2.682 (2.549–2.854)	2.529 (2.236–2.749)	0.1123	0.144
Putamen total volume %	0.535 (0.486–0.572)	0.491 (0.457–0.538)	0.1259	0.144
Thalamus total volume %	0.734 (0.658–0.774)	0.687 (0.620–0.736)	0.2854	0.285

Abbreviations: SFG, superior frontal gyrus; FDR, false discovery rate. Note: Volumes are expressed as percentages of intracranial volume. *p*-values were adjusted for multiple comparisons using the Benjamini–Hochberg false discovery rate (FDR) procedure.

**Table 3:** Receiver operating characteristic (ROC) analysis of individual MRI volumetric and inflammatory biomarkers for differentiating progressive supranuclear palsy (PSP) from Parkinson’s disease (PD).

Biomarker	Direction for PSP	AUC	95% CI	*p*	FDR q	Cut-Off	Sensitivity	Specificity
SFG total volume %	lower	0.927	0.808–1.000	<0.0001	<0.001	≤1.885	83.3%	100.0%
Pallidum total volume %	lower	0.885	0.749–1.000	<0.0001	<0.001	≤0.154	75.0%	100.0%
Frontal total volume %	lower	0.757	0.561–0.953	0.0103	0.0412	≤11.164	75.0%	75.0%
Caudate total volume %	lower	0.750	0.548–0.952	0.0151	0.0453	≤0.456	66.7%	83.3%
Brainstem volume %	lower	0.729	0.526–0.933	0.0272	0.0653	≤1.214	50.0%	91.7%
NEU	higher	0.715	0.506–0.925	0.0438	0.0876	3.1	100.0%	41.7%
NLR	higher	0.715	0.498–0.932	0.0521	0.0893	≥2.515	58.3%	91.7%
SII	higher	0.681	0.463–0.898	0.1043	0.1565	≥553.056	58.3%	75.0%
RDW-CV	higher	0.646	0.418–0.874	0.1914	0.2522	≥12.6	75.0%	58.3%
SIRI	higher	0.649	0.425–0.873	0.2102	0.25522	≥1.864	33.3%	100.0%
MLR	lower	0.563	0.323–0.802	0.6087	0.6640	≤0.302	58.3%	66.7%
PLR	higher	0.465	0.225–0.706	0.7773	0.7773	≥125.694	66.7%	50.0%

Abbreviations: AUC, area under the curve; CI, confidence interval; ROC, receiver operating characteristic; NEU, neutrophil count; NLR, neutrophil-to-lymphocyte ratio; MLR, monocyte-to-lymphocyte ratio; PLR, platelet-to-lymphocyte ratio; SIRI, systemic inflammation response index; SII, systemic immune-inflammation index; SFG, superior frontal gyrus. Note: ROC *p*-values test the null hypothesis of AUC = 0.5 and should not be interpreted as *p*-values for direct between-group comparisons. Optimal cut-off values were determined using the Youden index. Sensitivity and specificity are presented for the optimal threshold. *p*-values obtained from ROC analyses were adjusted for multiple comparisons using the Benjamini–Hochberg false discovery rate (FDR) procedure across all 12 biomarkers presented in the table.

**Table 4:** Diagnostic performance of logistic regression models combining MRI volumetric and peripheral inflammatory biomarkers for differentiating progressive supranuclear palsy (PSP) from Parkinson’s disease (PD).

Model	Predictors	AUC	Bootstrap Optimism	Optimism-Corrected AUC	95% CI	Cut-Off	Sensitivity (%)	Specificity (%)	Accuracy (%)
Blood biomarker	NLR	0.715			0.498–0.932	2.515	58.3	91.7	75.0
MRI biomarker	Pallidum total volume %	0.885			0.749–1.000	0.154	75.0	100.0	87.5
Combined primary model	Pallidum total volume % + NLR	0.903	0.024	0.879	0.771–1.000	0.771	75.0	100.0	87.5

Abbreviations: AUC, area under the receiver operating characteristic curve; CI, confidence interval; NLR, neutrophil-to-lymphocyte ratio; PD, Parkinson’s disease; PSP, progressive supranuclear palsy. Note: Cut-off values for combined models refer to predicted probabilities derived from binary logistic regression. Optimal thresholds were determined using the Youden index. Areas under the receiver operating characteristic curve (AUC), optimal cut-off values, sensitivity, specificity and overall accuracy are presented. Comparison of the MRI biomarker with the combined primary model: ΔAUC 0.02, DeLong *p* = 0.732. For the combined pallidum–NLR model, the cut-off value of 0.771 represents the predicted probability of PSP generated by the logistic regression model.

**Table 5:** Logistic regression model combining normalized pallidal volume and neutrophil-to-lymphocyte ratio for differentiating progressive supranuclear palsy from Parkinson’s disease.

Predictor	β	SE	OR	95% CI	*p*
Intercept	5.786	4.257	—	—	0.174
Pallidum total volume, per 0.01% ICV increase	−0.593	0.254	0.553	0.336–0.910	0.0198
NLR	1.787	1.016	5.97	0.81–43.75	0.0788

Abbreviations: CI, confidence interval; ICV, intracranial volume; NLR, neutrophil-to-lymphocyte ratio; OR, odds ratio. Note: PSP was coded as the positive outcome. For interpretability, the pallidal volume coefficient and OR are presented per 0.01 percentage-point increase in normalized pallidal volume.

## Data Availability

The datasets generated and/or analyzed during the current study are available from the corresponding author upon reasonable request.

## Supplementary Materials

The following supporting information can be downloaded at https://www.mdpi.com/article/10.3390/diseases14090330/s1. Table S1: Diagnostic performance of pallidal volumetry, NLR, and the combined pallidum–NLR model for differentiating PSP from PD.
