# Supplementary material for: Pallidal Volumetry and Neutrophil-to-Lymphocyte Ratio in Differentiating Progressive Supranuclear Palsy from Parkinson’s Disease
Source: Diseases. 2026 Sep 9;14(9):330. doi: 10.3390/diseases14090330 (PMC13605819; doi:10.3390/diseases14090330)
Supplement: Supplementary file 1 [file diseases-14-00330-s001.zip › diseases-4521671-supplementary.pdf]

Table S1. Diagnostic performance of pallidal volumetry, NLR, and the combined pallidum–NLR model for differentiating PSP from PD.

**Biomarker/model**	**Cut-off**	**TP**	**FN**	**TN**	**FP**	**Sensitivity, % (95% CI)**	**Specificity, % (95% CI)**	**Accuracy, % (95% CI)**
Pallidum total volume %	≤0.154	9	3	12	0	75.0 (42.8–94.5)	100.0 (73.5–100.0)	87.5 (67.6–97.3)
NLR	≥2.515	7	5	11	1	58.3 (27.7–84.8)	91.7 (61.5–99.8)	75.0 (53.3–90.2)
Pallidum + NLR	≥0.771	9	3	12	0	75.0 (42.8–94.5)	100.0 (73.5–100.0)	87.5 (67.6–97.3)

Note: Cut-off values were determined using the Youden index and represent exploratory cohort-derived thresholds. Sensitivity, specificity, and accuracy are apparent in-sample estimates. Ninety-five percent confidence intervals were calculated using the exact binomial (Clopper–Pearson) method. TP, true positive; FN, false negative; TN, true negative; FP, false positive; NLR, neutrophil-to-lymphocyte ratio.
